# Simultaneous saccharification and co-fermentation for bioethanol production using corncobs at lab, PDU and demo scales

**DOI:** 10.1186/1754-6834-6-2

**Published:** 2013-01-14

**Authors:** Rakesh Koppram, Fredrik Nielsen, Eva Albers, Annika Lambert, Sune Wännström, Lars Welin, Guido Zacchi, Lisbeth Olsson

**Affiliations:** 1 Department of Chemical and Biological Engineering, Industrial Biotechnology, Chalmers University of Technology, Göteborg SE-412 96, Sweden; 2 Department of Chemical Engineering, Lund University, P.O. Box 124, Lund, SE-221 00, Sweden; 3 Taurus Energy AB, Ideon, Ole Römers väg 12, Lund, SE-223 70, Sweden; 4 SEKAB E-Technology AB, P.O. Box 286, Örnsköldsvik, SE-891 26, Sweden

**Keywords:** *S*. *cerevisiae*, SSCF, Prefermentation, Xylose

## Abstract

**Background:**

While simultaneous saccharification and co-fermentation (SSCF) is considered to be a promising process for bioconversion of lignocellulosic materials to ethanol, there are still relatively little demo-plant data and operating experiences reported in the literature. In the current work, we designed a SSCF process and scaled up from lab to demo scale reaching 4% (w/v) ethanol using xylose rich corncobs.

**Results:**

Seven different recombinant xylose utilizing *Saccharomyces cerevisiae* strains were evaluated for their fermentation performance in hydrolysates of steam pretreated corncobs. Two strains, RHD-15 and KE6-12 with highest ethanol yield and lowest xylitol yield, respectively were further screened in SSCF using the whole slurry from pretreatment. Similar ethanol yields were reached with both strains, however, KE6-12 was chosen as the preferred strain since it produced 26% lower xylitol from consumed xylose compared to RHD-15. Model SSCF experiments with glucose or hydrolysate feed in combination with prefermentation resulted in 79% of xylose consumption and more than 75% of the theoretical ethanol yield on available glucose and xylose in lab and PDU scales. The results suggest that for an efficient xylose conversion to ethanol controlled release of glucose from enzymatic hydrolysis and low levels of glucose concentration must be maintained throughout the SSCF. Fed-batch SSCF in PDU with addition of enzymes at three different time points facilitated controlled release of glucose and hence co-consumption of glucose and xylose was observed yielding 76% of the theoretical ethanol yield on available glucose and xylose at 7.9% water insoluble solids (WIS). With a fed-batch SSCF in combination with prefermentation and a feed of substrate and enzymes 47 and 40 g l^-1^ of ethanol corresponding to 68% and 58% of the theoretical ethanol yield on available glucose and xylose were produced at 10.5% WIS in PDU and demo scale, respectively. The strain KE6-12 was able to completely consume xylose within 76 h during the fermentation of hydrolysate in a 10 m^3^ demo scale bioreactor.

**Conclusions:**

The potential of SSCF is improved in combination with prefermentation and a feed of substrate and enzymes. It was possible to successfully reproduce the fed-batch SSCF at demo scale producing 4% (w/v) ethanol which is the minimum economical requirement for efficient lignocellulosic bioethanol production process.

## Background

The global CO_2_ emissions in 2010 from fossil energy use grew at the fastest rate since 1969. The year 2010 also witnessed that the global oil production did not match the rapid growth in consumption [1]. These recent data further intensify worldwide concerns about greenhouse gas emissions and energy security for a sustained economic development. For a reduced dependence on oil from fossil reserves, use of biofuels such as bioethanol from abundantly available lignocellulosic biomass is of great interest nowadays because they will count towards meeting the mandate of 10% binding target for biofuels from renewable sources in the transport for all European member states by 2020 [2]. Along with this interest comes increased interest in commercializing ethanol production technology from inexpensive lignocellulosic feedstocks which includes wood biomass, agricultural and forestry residues, biodegradable fraction of industrial and municipal wastes. Irrespective of type, the basic structural composition of lignocellulosic biomass consists of cellulose, hemicellulose and lignin. The cellulose and hemicellulose that form the polysaccharide fraction are embedded in a recalcitrant and inaccessible arrangement [3] and therefore requires a pretreatment step to disrupt the structure and make it accessible for subsequent steps. Since lignocellulosic materials are very complex, not one pretreatment method can apply for all the materials. Several methods that are classified in to physical, physico-chemical, chemical and biological pretreatment have been investigated and an elaborate review on each of these methods has been presented by Taherzadeh and Karimi [4]. One of the most commonly used pretreatment methods is steam explosion, with the addition of H_2_SO_4_ or SO_2_, which removes most of the hemicellulose, followed by enzymatic hydrolysis to convert cellulose to glucose [5,6].

The release of hexose and pentose sugars during pretreatment and enzymatic hydrolysis is often accompanied by liberation of compounds such as furans, weak organic acids and phenolics compounds [7] that inhibits growth, ethanol yield and productivity of fermenting microorganism, *Saccharomyces cerevisiae*[8-10]. Traditionally and industrially relevant microorganism for ethanol fermentation is *S. cerevisiae*, but its inability to consume pentose sugars like xylose and arabinose has led to intensive research on metabolic and evolutionary engineering to develop strains that can tolerate high concentration of inhibitors and ferment xylose and arabinose [11-15]. However, it has been shown that recombinant *S. cerevisiae* strain utilizing pentose sugar may lose its xylose consuming ability in a long term evolutionary engineering for inhibitor tolerance [15]. Consequently, to ensure that all properties are retained during evolutionary engineering requires careful design of the selection pressure.

The enzymatic hydrolysis can be performed simultaneously with the co-fermentation of glucose and xylose in a process referred to as simultaneous saccharification and co-fermentation (SSCF). Besides reduced capital cost [16], SSCF process offers several advantages which include continuous removal of end-products of enzymatic hydrolysis that inhibit cellulases or β-glucosidases [17] and higher ethanol productivity and yield than separate hydrolysis and fermentation [18,19]. It is required to operate a SSCF process at high content of water-insoluble-solids (WIS) to achieve high concentrations of ethanol. However, it has been shown that at high WIS content ethanol yield was decreased due to increased mass transfer resistance and inhibitors concentration [20]. Operating SSCF in a fed-batch mode at high WIS content not only assists ease of mixing and produces high ethanol concentrations [21] but also offers a possibility to maintain glucose at low levels allowing efficient co-fermentation of glucose and xylose [22]. Lowering of glucose concentration can be achieved by initially fermenting free hexoses before adding enzymes to a SSCF process in a concept referred as prefermentation enhanced xylose uptake irrespective of batch or fed-batch SSCF [23]. These flexibilities offered by a SSCF process makes it a promising process option for bioethanol production from lignocellulosic materials.

The heterogeneity of raw materials together with a variety of pretreatment methods, lack of detailed understanding of dynamic changes of substrate during enzymatic hydrolysis and unavailability of microorganisms that can ferment a wide range of carbohydrates and can tolerate high concentrations of inhibitors produced from pretreated biomass makes SSCF a highly researched area yet to reach the commercial status. There come additional technical challenges when operating at larger scales which include longer times to add material into the reactor, longer mixing times and therefore concentration gradients are inevitable. On-site propagation of yeast in large volumes is needed which also increases the probability of contamination since lignocellulosic ethanol plants will not employ aseptic operating conditions. Moving cellulosic ethanol technology from the laboratory to a commercial scale biorefinery is an expensive proposition and requires process data at sufficient scale to obtain engineering and process guarantees. Some prominent players that are working on this proposition include Chemtex, Inbicon, DuPont cellulosic ethanol, POET-DSM advanced biofuels, Iogen, Abengoa Bioenergy, Mascoma and SEKAB. A category of feedstock that is of considerable interest is corn derived residues due to that it is inexpensive and available in abundance. Corncob is an agricultural residue and a byproduct of corn production. Currently, 12.1 billion tons and 120 million tons of corn are being produced in the US and China, respectively. About 70 million metric tons of corncobs are available annually accounting only from the US and China markets [21,24]. Removal of corncobs from the agricultural grounds does not contribute to decreased soil organic matter since corncobs are low in nutrients.

In this work, a xylose fermenting *S. cerevisiae* strain was used in SSCF of pretreated corncobs with the objective of determining suitable conditions for co-consumption of glucose and xylose. Fed-batch mode of SSCF in combination with prefermentation was investigated at high WIS content. To validate the designed SSF process and verify the reproducibility at different scales, the process was scaled up from lab conditions to process development unit (PDU) (30 liters) and further to demo scale (10 m^3^).

## Results and discussion

The SSCF concept is one of the interesting process options and the potential of such process for biological conversion of lignocellulosic raw materials to bioethanol in large scales has to the best of our knowledge not been reported previously. A promising xylose consuming strain of *S. cerevisiae* was selected from screening seven different recombinant *S. cerevisiae* strains. The glucose influence on xylose consumption of the selected strain was investigated by model SSCF with glucose or hydrolysate feed. The potential of fed-batch SSCF process in combination with prefermentation was finally demonstrated in 10 m^3^ demo scale bioreactors.

### Screening and selection of *S. cerevisiae* strain

#### Anaerobic fermentation of corncob hydrolysate

**Figure 1:**
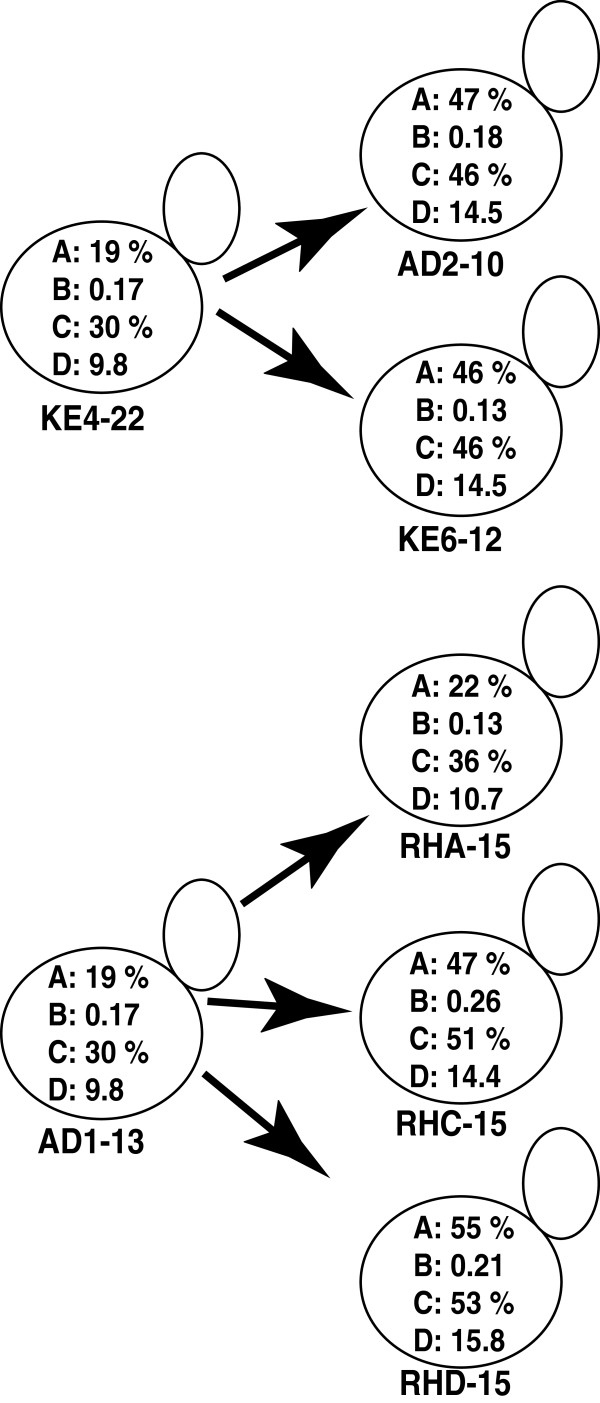
**Screening of *S. cerevisiae *strains in corncobs hydrolysate.** Xylose consumption, xylitol and ethanol yields, ethanol concentration in corncobs hydrolysate after 96 h of fermentation in anaerobic shake flasks. KE4-22 is the parental strain of AD2-10 and KE6-12. AD1-13 is the parental strain of RHA-15, RHC-15 and RHD-15. A: xylose consumed (%), B: xylitol yield on consumed xylose (g g^-1^), C: ethanol yield (%, based on maximum theoretical ethanol yield on available glucose and xylose), D: ethanol concentration (g l^-1^) at the end of 96 h.

The seven different *S. cerevisiae* strains (Table 1) were evaluated on their fermentation performance in corncobs hydrolysate in shake flasks equipped with glycerol loops. Since, xylose constitutes a significant proportion of monosaccharides in corncobs hydrolysate xylose consumption and xylitol yield together with ethanol yield were determined (Figure 1) and used as parameters for strain selection. The strains, AD2-10, KE6-12 and RHC-15, RHD-15 displayed similar ethanol concentration, ethanol yield and performed better than their respective parental strains with regard to xylose consumption. The strain RHD-15 displayed the highest ethanol yield and xylose consumed. The strain KE6-12 stands alone among other strains in xylitol yield producing the lowest amount of xylitol from consumed xylose. Even though the screening revealed significant differences in fermentation of hydrolysate, it is important to evaluate the microbial performance in the whole slurry in a SSCF process. The strains RHD-15 and KE6-12, due to their high ethanol and low xylitol yields, were therefore, selected as the preferred strains for subsequent investigations in the SSCF process.

**Table 1:** ***S cerevisiae *strains used in this study**

**Parental strain**	**Evolved strain**
KE4-22	AD2-10
	KE6-12
AD1-13	RHA-15
	RHC-15
	RHD-15

#### SSCF of corncobs whole slurry

**Figure 2:**
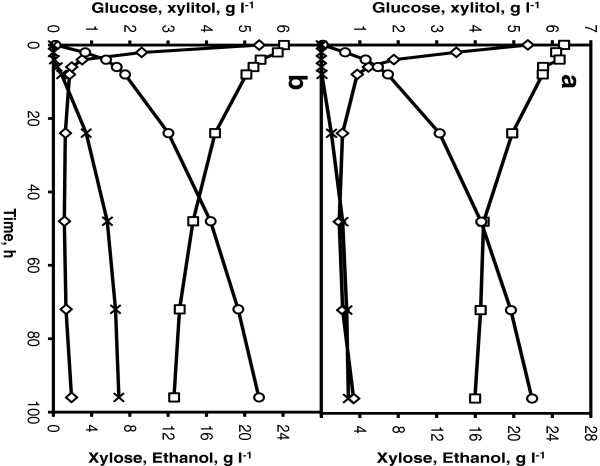
**Screening of *S. cerevisiae *strains in corncobs whole slurry.** Glucose (diamonds) and xylose (squares) consumption, ethanol (circles) and xylitol (crosses) production in SSCF at 7.5% WIS content, 5 g l^-1^ of yeast loading and 5 FPU gWIS^-1^ of enzyme loading using KE6-12 (**a**) and RHD-15 (**b**).

To assess the fermentation performance, the strains RHD-15 and KE6-12 were evaluated in a base case batch SSCF of corncobs whole slurry at 7.5% WIS for ethanol production. During the SSCF process, the glucose concentration was quickly reduced to less than 1 g l^-1^ within 10 h and thereafter, it was maintained at this level throughout the process (Figure 2a &2b). Immediately after inoculation, both the strains started to consume xylose for a period of 72 h after which the xylose concentration in the reactor started to level off. After 96 h, the strain KE6-12 had consumed 37% of the available xylose and 30% of the consumed xylose was converted to xylitol (2.8 g l^-1^) whereas, the strain RHD-15 had consumed 42% of the available xylose and 66% of the consumed xylose was converted to xylitol (6.4 g l^-1^). An ethanol concentration of 21.9 and 21.5 g l^-1^ were achieved corresponding to a yield of 0.28 g g^-1^ and 0.27 g g^-1^ based on total available sugars for the strains KE6-12 and RHD-15, respectively. In comparison to RHD-15, strain KE6-12 consumed marginally lower amount of xylose but, produced 56% less xylitol. Since, bioconversion of xylose to ethanol is one of the predominant requirements for an economical lignocellulosic bioethanol production, further fermentation and SSCF experiments were carried out with the strain KE6-12, unless otherwise stated. In screening experiments using corncobs hydrolysate, the strain RHD-15 performed relatively better than KE6-12, however, in SSCF using corncobs whole slurry both the strains resulted in similar ethanol yields and RHD-15 was clearly outperformed by KE6-12 in lower xylitol yields. The differences in results from the two screening experimental systems could be attributed to the differences in experimental conditions. The effect of pH on xylose consumption by a recombinant xylose utilizing *S. cerevisiae* has been previously shown that increasing the pH from 5.0 to 5.5 resulted in 46% increase in xylose consumption rate [25]. Screening using corncobs hydrolysate in shake flasks were performed at an initial pH 6.0 and 30°C which clearly resulted in higher xylose consumption compared to screening in SSCF where the pH was controlled at 5.0 and sub-optimal temperature of 35°C. It should be noted that often strain engineering and development results in a numerous strains and a high throughput screening of these strains in SSCF process in shake flasks could be impractical due to difficulties in mixing at high WIS content. The difference in two screening systems illustrate the importance of choice of experimental setup and conditions for screening to be as close as possible to that used in the actual experiments.

### Model SSCF as a tool to design the SSCF process

In order to understand the effect of glucose on xylose consumption and to optimally design the SSCF process with effective xylose consumption a model SSF [26] with prefermentation [23] was performed. A model SSCF is a SSCF process without the addition of enzymes but fed with pure glucose solution or hydrolysate to the reactor mimicking the release of glucose during enzymatic hydrolysis of cellulose. Prefermentation is a concept where initially available free glucose was fermented before starting the feed.

#### Lab scale

**Figure 3:**
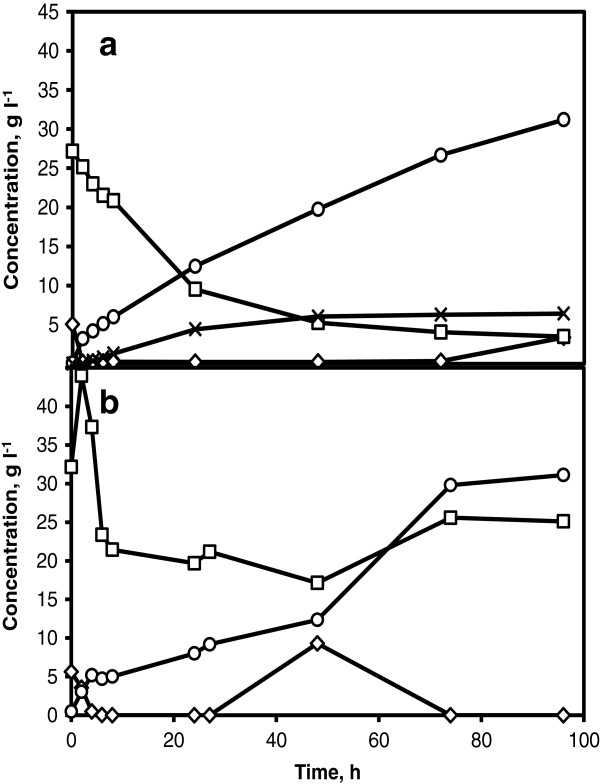
**Model SSCF.** Glucose (diamonds) and xylose (squares) consumption, ethanol (circles) and xylitol (crosses) production in a model SSCF in corncobs hydrolysate with 5 g l^-1^ of KE6-12 at lab scale using a feed of glucose solution (**a**) and at PDU using a feed of liquid fraction after enzymatic hydrolysis (**b**). Amount of glucose fed is corresponding to 7.5% WIS content.

Model SSCF in lab scale was performed in corncobs hydrolysate with a feed of 100 g l^-1^ glucose solution at a constant rate. A glucose feed corresponding to the amount of glucose from 7.5% WIS was started after 2 h of inoculation and terminated at 96 h. During the prefermentation period of 2 h, the glucose concentration was reduced to nearly 0 g l^-1^ and maintained at this level until 72 h (Figure 3a). Immediately after prefermentation, xylose was rapidly consumed until 48 h and thereafter, the concentration started to level off. After 96 h, 79% of xylose was consumed and 37% of consumed xylose was converted to xylitol (6.4 g l^-1^). An ethanol concentration of 31.2 g l^-1^ was achieved corresponding to a yield of 0.38 g g^-1^ based on total available sugars (75% of the theoretical yield). Higher ethanol concentration in model SSCF compared to batch SSCF may possibly be due to higher xylose consumption and also points to a direction that cellulose fibers were not completely hydrolyzed in batch SSCF to yield similar ethanol concentrations as that obtained in model SSCF.

#### *PDU* scale

A model SSCF in PDU scale similar to lab scale model SSCF was performed with a feed of hydrolysate from enzymatic hydrolysis. In order to completely hydrolyze cellulose fibers, enzymatic hydrolysis of solid fraction of corncobs slurry was carried out at 50°C with enzyme loading of 6 FPU gWIS^-1^. The liquid fraction remaining after filtering the enzymatically hydrolyzed solid fraction was used as a feed. Prefermentation in corncobs hydrolysate was initiated by adding yeast and an enzyme solution corresponding to 3 FPU gWIS^-1^. The glucose was rapidly consumed during the initial 10 h of prefermentation, reduced to near 0 g l^-1^ and maintained at this level until 24 h (Figure 3b). A sharp increase in xylose concentration was observed immediately after the addition of enzyme solution indicating the hydrolysis of xylan. Thereafter, the xylose was consumed quickly for 10 h, however, when the glucose was completely consumed the xylose consumption dramatically slowed down. This indicates that the consumption of glucose with maintained low concentration of glucose is beneficial for efficient xylose consumption. Previous study on fed-batch SSCF using xylose rich wheat straw has highlighted that maintaining low levels of glucose consequently increased consumed xylose twice as compared to a batch SSCF [26]. It also has been discussed that presence of glucose at high concentrations may inhibit xylose uptake due to competition for transporters [27,28]. Feeding of the liquid fraction from enzymatic hydrolysis was started after 24 h of prefermentation and was maintained for 24 h corresponding to a final WIS content of 7.5%. The glucose concentration gradually increased during the 24 h feeding phase until 48 h and thereafter was completely consumed. The xylose started to accumulate when glucose concentration reached a peak of 10 g l^-1^ and thereafter no xylose was consumed and no change in ethanol concentration was observed indicating the end of fermentation. The increase in xylose concentration after 50 h could be due to enzymatic hydrolysis of xylan. After 96 h, an ethanol concentration of 32 g l^-1^ was produced corresponding to 77% of the theoretical yield based on available sugars. This ethanol yield is well in accordance with ethanol yields of model SSCF in lab scale. Evidences from model SSCF with prefermentation clearly suggest that fermentation of initial free glucose and thereafter, maintenance of glucose at low levels are crucial factors for efficient xylose consumption.

### Fed-batch SSCF

#### *PDU* scale

**Figure 4:**
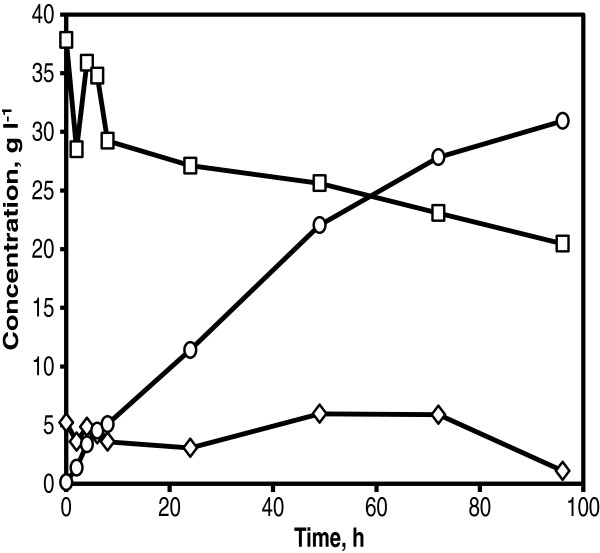
**Fed-batch SSCF with prefermentation and split addition of enzyme at PDU.** Glucose (diamonds) and xylose (squares) co-consumption, ethanol (circles) and xylitol (crosses) production using corncobs whole slurry at 7.9% WIS, 6 g l^-1^ of KE6-12 with 3 FPU gWIS^-1^ of enzyme loading at each time points of 2 h, 24 h and 48 h.

It was also possible to achieve similar ethanol yields in a fed-batch SSCF as that in the model SSCF. Fed-batch SSCF in PDU was carried out using the whole slurry with a total WIS of 7.9%. Initially, prefermentation was carried out for 2 h by adding 6 g dry cell weight l^-1^ of yeast from cell suspension. To maintain glucose concentrations at a minimum level in the reactor and thereby facilitate effective xylose consumption, a strategy to add enzyme solution at multiple time points to ensure controlled release of glucose was investigated. An enzyme solution corresponding to 3 FPU gWIS^-1^ was added at 2 h, 24 h, and 48 h. The glucose concentration was maintained around 5 g l^-1^ until 72 h before it was completely consumed at 96 h (Figure 4). A steady co-consumption of glucose and xylose was observed throughout the SSCF. After 96 h, 50% of the available xylose was consumed producing xylitol with a concentration of only 1.5 g l^-1^. An ethanol concentration of 32 g l^-1^ was achieved corresponding to 76% of the theoretical ethanol yield based on available sugars.

**Figure 5:**
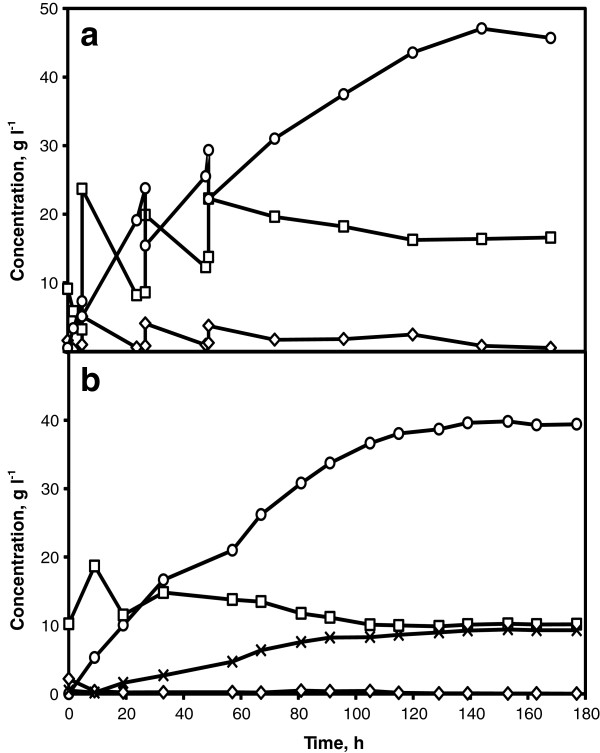
**SSCF with prefermentation and fed-batch addition of substrate and enzyme.** Glucose (diamonds) and xylose (squares) co-consumption, ethanol (circles) and xylitol (crosses) production using corncobs whole slurry at 10.5% WIS, 5 g l^-1^ of KE6-12 and 15 FPU gWIS^-1^ of enzyme loading. Split addition of substrate at 0 h, 5 h, 27 h, 49 h and enzyme solution at 2 h, 24 h, 48 h, 72 h, 96 h in PDU (**a**). Fed-batch addition of substrate for 48 h and split addition of enzyme solution at 2 h, 24 h, 48 h, 72 h, 96 h in demo scale (**b**).

In a commercial bioethanol production process it is desirable that the substrate load is higher than 7% WIS to achieve 4% (w/v) ethanol concentration to yield a subsequent economical distillation process [29]. It has been shown that working at high WIS content increases the concentration of inhibitors and results in inhibition of yeast and lower ethanol yields [26]. Therefore, along with split addition of enzymes, fed-batch SSCF at higher WIS was investigated with split addition of substrate resulting in lower amount of inhibitors for each addition. Fed-batch SSCF experiment was performed with a corncobs slurry addition at 0 h, 5 h, 27 h and 49 h to a total final WIS of 10%. Enzyme solution was added at multiple time points of 2 h, 24 h, 48 h, 72 h and 96 h to a total of 15 FPU gWIS^-1^. During the first 2 h of prefermentation, the glucose concentration was reduced to nearly 0 g l^-1^ and reached around 5 g l^-1^ after the first addition of enzyme (Figure 5a). The glucose concentration was then maintained below 5 g l^-1^ throughout the SSCF process. The xylose was co-consumed along with glucose for more than 100 h. At the end of fed-batch SSCF, 55% of the available xylose was consumed and 11% of the consumed xylose was converted to xylitol (3.4 g l^-1^). An ethanol concentration of 47 g l^-1^ was achieved corresponding to a yield of 0.35 g g^-1^ based on total available sugars (69% of the theoretical yield). Higher xylose consumption and ethanol yield at 10% WIS clearly suggest that the combination of prefermentation and a feed of enzymes and substrates as one of the possible SSCF strategies for demo scale execution.

### Demo scale

#### Xylose fermentation in hydrolysate

**Figure 6:**
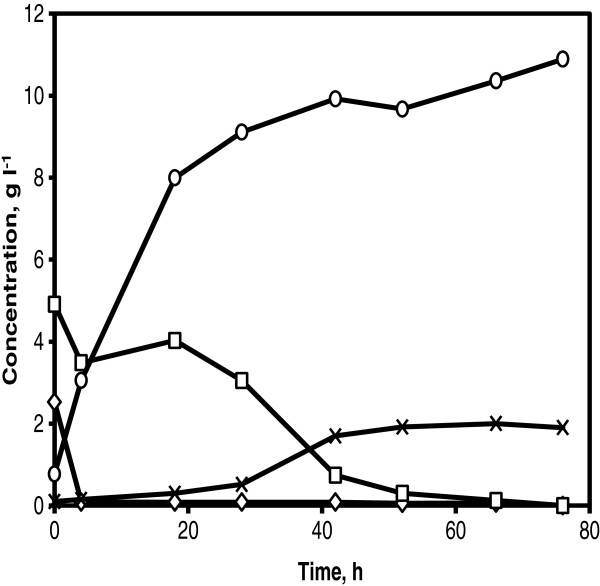
**Fed-batch fermentation in corncobs hydrolysate at demo scale.** Glucose (diamonds) and xylose (squares) consumption, ethanol (circles) and xylitol (crosses) production using 5 g l^-1^ of KE6-12. Corncobs hydrolysate corresponding to 6% WIS content was fed for 24 h.

A time span of 24 to 48 h was used to pump a substrate in to the demo scale reactor of 10 m^3^. In order to address the potential of the strain KE6-12 on xylose consumption, fed-batch fermentation of corncobs hydrolysate corresponding to a WIS content of 6% was evaluated. The corncobs hydrolysate was fed into the reactor for 24 h. The glucose concentration was reduced to nearly 0 g l^-1^ within 5 h and all xylose was consumed in 76 h (Figure 6). Only 10.6% of the consumed xylose was converted to xylitol (2.0 g l^-1^). An ethanol concentration of 10.9 g l^-1^ was achieved corresponding to a yield of 0.46 g g^-1^ on total available sugars (90% of the theoretical yield).

#### Fed-batch SSCF

A fed-batch SSCF with substrate and enzyme feed and prefermentation for 2 h similar to the one performed at PDU scale was carried out in the demo scale. The corncobs slurry was fed into the reactor for 48 h resulting in a total WIS of 10.5%. Enzyme solution was added at five different time points, 2 h, 24 h, 48 h, 72 and 96 h corresponding to a total of 15 FPU gWIS^-1^. The glucose concentration was quickly reduced to nearly 0 g l^-1^ within 10 h and thereafter, it was maintained at low concentration throughout the process (Figure 5b). Co-consumption of xylose and glucose was observed for more than 100 h similar to the fed-batch SSCF at PDU scale. After 150 h, 65% of the available xylose was consumed and 24.7% of the consumed xylose was converted to xylitol (9.3 g l^-1^). Surprisingly, in comparison to fed-batch SSCF at PDU scale, higher amount of xylose was consumed in demo scale, however, also higher amount of xylitol was produced. An ethanol concentration of 39.8 g l^-1^ was achieved corresponding to a yield of 0.29 g g^-1^ based on total available sugars (58% of the theoretical yield). More controlled conditions of temperature, pH and homogenous mixing in PDU scale resulted in higher final ethanol concentration and yield compared to demo scale conditions with higher mass transfer limitations.

## Conclusion

The performance of recombinant xylose utilizing *S. cerevisiae* strains varied in two different screening experiments, which highlights the importance of experimental setup and conditions for screening of strains to be highly similar to that of the actual experiments. The choice of the strain KE6-12 seems well justified when xylose was completely consumed at demo scale during the fermentation of hydrolysate with 90% of the theoretical ethanol yield. Different feeding profiles of glucose and its influence on xylose consumption was studied using model SSCF and it proved to be a valuable tool to optimally design a SSCF process. The potential of the fed-batch SSCF process is more vivid and we demonstrated that with prefermentation and substrate and enzyme feed it is possible to produce ethanol from corncobs as high as 40 g l^-1^ and more, with relatively high WIS content at both 30 l (PDU scale) and 10 m^3^ (demo scale). Using such a strategy it was possible to maintain low levels of glucose concentration, which facilitated co-consumption of glucose and xylose. We also confirmed that the results of fed-batch SSCF were similar at PDU and demo scales and the experimental system was reproducible at both the scales. However, at higher WIS content an optimal feeding strategy is required to ferment all xylose and avoid glucose accumulation.

## Materials and methods

### *Saccharomyces cerevisiae* strains

The seven *S. cerevisiae* strains used in this study (Table 1) were developed by a combination of different evolutionary engineering strategies and random mutagenesis (Albers *et al.*, manuscript in preparation) on *S. cerevisiae* TMB 3400 [30] that harbours the xylose reductase gene and xylitol dehydrogenase from *Scheffersomyces stipitis* (formerly known as *Pichia stipitis*) and endogenous xylulokinase overexpressed. All the strains were stored at −80°C in culture aliquots containing 20% sterile glycerol. Volumes of 100 μl from the vials were used to inoculate precultures.

### Media

#### Corncobs slurry

Corncobs slurry with a water-insoluble-solids (WIS) content of 15% was received from SEKAB-E-Technology AB (Örnsköldsvik, Sweden) and was stored at −20°C. The corncobs were pretreated at 185°C for 5 min with 0.6% dilute sulfurous acid (SO_2_ in water). Two batches were pretreated and the composition of which are presented in the Table 2. Batch 1 was used for screening and selection experiments. Batch 2 was used in the demo scale experiments. The corncobs hydrolysate (liquid fraction of corncobs slurry), pH adjusted to 5.0 with 10 M NaOH, was used in yeast cell cultivations when required.

**Table 2:** Composition of the pretreated corncobs

**Content in solid fraction (% of WIS)**			**Content in liquid fraction (g l**^**-1**^**)**		
	**Batch 1**	**Batch 2**		**Batch 1**	**Batch 2**
Glucan	66.9	61.4	Glucose^*^	17.0	15.0
Mannan	0	0	Mannose^*^	0	0
Galactan	0	2.9	Galactose^*^	6.5	0
Xylan	5.8	8.2	Xylose^*^	79.4	74.4
Arabinan	1.0	1.4	Arabinose^*^	11.8	14.0
Lignin	27.6	28.9	HMF	2.0	1.9
			Furfural	3.8	4.0
			Acetic acid	10.4	8.3

^*^Both monomeric and oligomeric form is included.

#### Molasses

Molasses was obtained from SEKAB-E-Technology AB (Örnsköldsvik, Sweden) and was either used alone or mixed with liquid fraction of corncobs slurry for cultivating yeast cells that was then used for SSCF experiments.

#### Minimal medium

The initial inoculum for screening yeast strains and SSCF experiments were cultivated in minimal medium containing 20 g l^-1^ glucose and xylose, respectively and enriched with salts, two folds of vitamins and trace elements according to Verduyn *et al.*[31]. The pH of the medium was set to 6.0 with 1 M NaOH for all shake flask cultivations.

### Cultivation of yeast

In order to improve inhibitor tolerance by adaptation, yeast cells were grown briefly in presence of corncobs hydrolysate during the cultivation for screening and SSCF experiments (as described below). It has been previously shown that the cultivation procedure of yeast significantly influences the performance in SSF and small-scale fermentations of hydrolysate liquor [32].

The precultures for screening *S. cerevisiae* strains for ethanol production were cultivated in 150 ml shake flasks with 50 ml of minimal medium. The cultures were inoculated to an initial OD_650_ of 0.005, incubated at 30°C on an orbital shaker at 180 rpm. After 18 h of incubation, corncobs hydrolysate supplemented with 23.5 g l^-1^ (NH_4_)_2_SO_4_, 3.0 g l^-1^ KH_2_PO_4_ and 2.25 g l^-1^ MgSO_4_ · 7H_2_O was added to the preculture cultivation flask to a final volume of 35% (v/v) and incubated for another 24 h.

The yeast cells for SSCF experiments in lab and PDU scales were cultivated in aerobic batch on molasses, followed by an aerobic fed batch on corncobs hydrolysate and molasses. In the demo scale molasses was used as the medium in aerobic batch and fed batch cultivation. The yeast strain was inoculated in to 50 ml (lab scale), 150 ml (PDU) of minimal medium contained in a 150 ml (lab scale) and 300 ml (PDU) shake flasks, respectively; incubated at 30°C on an orbital shaker at 180 rpm for 24 h. Aerobic batch cultivation was performed in 50 g l^-1^ molasses supplemented with 23.5 g l^-1^ (NH_4_)_2_SO_4_, 3.0 g l^-1^ KH_2_PO_4_, 2.25 g l^-1^ MgSO_4_ · 7H_2_O, 33 μg l^-1^ biotin, 125 ppm vitahop (Betatech Gmbh, Schwabach, Germany) (to suppress bacterial growth) and 0.5 ml l^-1^ antifoam. The yeast cultivation was carried out in 3.6 l Infors HT-Labfors bioreactor (lab scale), 30 l bioreactor (PDU) and 10 m^3^ bioreactor (demo scale). The cultivation was initiated in the bioreactors by adding 50 ml or 150 ml of minimal medium culture to a working volume of 500 ml (lab scale) or 1.5 l (PDU) of molasses medium, respectively. The cultivation was carried out until all sugars are consumed which was indicated by CO_2_ evolution in the gas-out and dissolved oxygen concentration in the culture. Upon depletion of sugars in batch phase, a feed solution containing corncobs hydrolysate and molasses was fed linearly for 20 h to a final volume of 1.5 l (lab scale) or 4.5 l (PDU). The concentration of corncobs hydrolysate in the feed solution was same as that of concentration of corncobs slurry in the SSCF experiments. Molasses concentration was 100 g l^-1^ in the feed solution. The stirrer speed during the batch phase in lab scale was 700 rpm and increased linearly to 1000 rpm during the fed batch phase; whereas, the stirrer speed was maintained at 700 rpm throughout the cultivation in PDU scale; aeration rate was maintained at 1 vvm and the pH was maintained at 5.0 by automatic addition of 2 M NaOH.

After the cultivation, cells were harvested by centrifugation for 8 min at 4°C, 1800 g and the cell pellet was resuspended in 0.9 % sterile NaCl solution to yield a cell suspension with a dry weight of 80 g l^-1^.

#### Anaerobic fermentation in shake flasks

The pH of corncobs hydrolysate was set to 6.0, supplemented with 0.5 g l^-1^ (NH_4_)_2_HPO_4_, 125 ppm vitahop and filter sterilized using 0.45 μm cellulose acetate filter. This fermentation medium was inoculated using the cell suspension to reach a yeast concentration of 3 g dry cell weight l^-1^. The fermentations were carried out in 50 ml working volume in 100 ml shake flasks fitted with glycerol loops providing anaerobic condition. The flasks were incubated at 30°C on an orbital shaker at 180 rpm for 96 h and samples were withdrawn for OD_650_ measurement and extracellular metabolite analysis. Possible contamination during the shake flask fermentation was checked by ocular inspection in microscope.

### SSCF

The SSCF experiments were carried out in lab scale (3.6 l Infors HT-Labfors), PDU scale (30 l), and demo scale (10 m^3^) bioreactors with a total working weight of 1.5 kg, 20 kg and 4000 kg, respectively. In the lab and PDU scale experiments the corncobs slurry was pH adjusted to 5.0 with 10 M NaOH and supplemented with 0.5 g l^-1^ (NH_4_)_2_HPO_4_. In the demo scale the pH was adjusted using NH_3_ solution and supplemented with 0.25 g l^-1^ H_3_PO_4_. To avoid possible contamination and foam formation 125 ppm of Vitahop solution and 0.5 ml l^-1^ antifoam, respectively were added to the medium. In order to obtain the desired WIS content the supplemented medium was diluted with water and used for SSCF experiments. Unless otherwise stated, all the experiments were initiated by adding 5 g dry cell weight l^-1^ of yeast from cell suspension. An enzyme preparation, Cellic Ctec-2 from Novozymes A/S, Denmark with filter paper activity of 95 FPU g^-1^ enzyme, β-glucosidase activity of 590 IU g^-1^ enzyme was added to SSCF experiments corresponding to the desired cellulase activity. All SSCF experiments were carried out at 35°C; pH was maintained at 5.0 by automatic addition of 3 M NaOH and the stirrer speed was maintained at 400 rpm in lab and PDU scales, respectively. A brief summary of all SSCF experiments carried out is listed in the Table 3. All SSCF experiments performed in duplicates in lab scale and one of them is represented in the results and discussion section.

**Table 3:** Brief list of SSCF experiments carried out in lab, PDU and demo scales

**Mode of operation_Scale**	**Initial Pre-fermentation time, h**	**Amount of solids, %WIS**	**Strain**	**Total cell amount, g l**^**-1**^	**Total enzyme amount, FPU gWIS**^**-1**^
Batch SSCF_Lab	None	7.5	RHD-15	5	5
Batch SSCF_Lab	None	7.5	KE6-12	5	5
Fed-batch Model SSCF_Lab^1^	2	7.5	KE6-12	5	None
Fed-batch Model SSCF_PDU^2^	24	7.5	KE6-12	5	None^*^
Fed-batch SSCF_PDU	2	7.9	KE6-12	6	9
Fed-batch SSCF_PDU	2	10	KE6-12	5	15
Fed-batch SSCF_Demo	2	10.5	KE6-12	5	15

^1^Model SSCF in lab scale with a feed of glucose solution with glucose amounts corresponding to 7.5%WIS.

^2^Model SSCF in PDU scale with a feed of filtered hydrolysate from enzymatic hydrolysis of whole slurry at 7.5% WIS.

^*^No enzyme added during the model SSCF. However, filtered hydrolysate from enzymatic hydrolysis of whole slurry using an enzyme solution of 6 FPU gWIS^-1^ was used as a feed solution.

### Analysis of metabolites

Samples for extracellular metabolites were analyzed by high performance liquid chromatography using Aminex HPX-87H column with 30 × 4.6 mm Cation-H Biorad micro-guard column maintained at 45°C. 5 mM H_2_SO_4_ was used as an eluent at a flow rate of 0.6 ml min^-1^. Ethanol, xylitol, and acetic acid were detected using RI detector maintained at 35°C and HMF, furfural and lactic acid were detected using UV detector at 210 nm. The sugars in corncobs hydrolysate and samples from shake flasks and SSCF experiments were analyzed by high performance anion exchange chromatography using 4 × 250 mm Dionex CarboPac PA1 column with 4 × 50 mm guard column maintained at 30°C. Eluent A: 300 mM NaOH, eluent B: 100 mM NaOH + 85 mM sodium acetate were used for elution at a flow rate of 1 ml min^-1^. Monosaccharides including arabinose, galactose, glucose, xylose and mannose were detected using pulsed amperometric detector. Optical density (OD) was used as an estimate of cell concentration in shake flask experiments. OD was measured at 650 nm using the cell free medium at the point of sampling as background.

### Yield calculations

#### Ethanol yield (% of maximum theoretical yield)

The sum of available fermentable sugars including glucose, mannose, galactose, and xylose in liquid fraction and glucan and xylan fibers in the WIS was calculated. Due to the addition of water during hydrolysis, the theoretical weight of glucose and xylose released are 1.11 and 1.13 times the weight of glucan and xylan, respectively. By using the maximum theoretical ethanol yield of 0.51 g g^-1^ sugar, the maximum ethanol that can be produced from total available sugars was calculated. The percentage of the theoretical ethanol yield is defined as Y _SE_ = 100*produced amount of ethanol (g)/maximum theoretical amount of ethanol (g).

#### Xylose consumed (%)

The percentage xylose consumed = 100*amount of xylose consumed (g)/total amount of available xylose in liquid and WIS fraction (g).

#### Xylitol yield (%)

The percentage xylitol yield = 100*amount of xylitol produced (g)/amount of xylose consumed (g).

## Abbreviations

SSCF: Simultaneous saccharification and co-fermentation; SSF: Simultaneous saccharification and fermentation; WIS: Water insoluble solids; PDU: Process development unit; FPU: Filter paper unit; OD: Optical density; HPLC: High performance liquid chromatography; HPAEC: High performance anion exchange chromatography.

## Competing interests

SW, AL were employed by SEKAB E-Technology during the time of this work. EA was employed by Taurus Energy AB during the time of this work. LW is employed by Taurus Energy AB and LO does consultancy for Taurus Energy AB.

## Authors’ contribution

RK, EA, AL, SW, LW, GZ and LO participated in the conception and design of the study. RK, AL and FN performed the experimental work. RK wrote the manuscript. All the authors commented on the manuscript, read and approved the final manuscript.
